# Influence of Membrane Asymmetry on OmpF Insertion, Orientation and Function

**DOI:** 10.3390/membranes13050517

**Published:** 2023-05-16

**Authors:** Annemarie Donoghue, Mathias Winterhalter, Thomas Gutsmann

**Affiliations:** 1 Research Center Borstel, Leibniz Lung Center, Parkallee 10, 23845 Borstel, Germany; 2 School of Science, Constructor University, Campus Ring 1, 28759 Bremen, Germany; 3 Center for Structural Systems Biology, Notkestraße 85, Building 15, 22607 Hamburg, Germany

**Keywords:** OmpF, lipopolysaccharide, asymmetry, antibiotic, enrofloxacin

## Abstract

The effect of asymmetric membranes containing lipopolysaccharides (LPS) on the outer membrane protein F (OmpF) reconstitution, channel orientation, and antibiotic permeation across the outer membrane was investigated. After forming an asymmetric planar lipid bilayer composed of LPS on one and phospholipids on the other side, the membrane channel OmpF was added. The ion current recordings demonstrate that LPS has a strong influence on the OmpF membrane insertion, orientation, and gating. Enrofloxacin was used as an example of an antibiotic interacting with the asymmetric membrane and with OmpF. The enrofloxacin caused the blockage of the ion current through the OmpF, depending on the side of addition, the transmembrane voltage applied, and the composition of the buffer. Furthermore, the enrofloxacin changed the phase behavior of the LPS-containing membranes, demonstrating that its membrane activity influences the function of OmpF and potentially the membrane permeability.

## 1. Introduction

The emergence of multidrug-resistant bacteria necessitates the search for new antibiotics or the improvement of existing antibiotics. Bacteria escape the toxic action of antibiotics in various ways. Among other mechanisms are the reduced permeability through the modulation of the outer membrane composition (number and modification of the outer membrane channels), enzymatic inactivation, and the production of efflux pumps [1]. In this study, we investigate the effect of lipopolysaccharides (LPS) on the function of the outer membrane protein F (OmpF).

Gram-negative bacteria are covered by an inner and an outer membrane separated by the periplasmic space. The first line of defense is the outer membrane, which has an asymmetric composition. The inner leaflet is composed of phospholipids, and the outer leaflet is composed of LPS. Water-filled membrane-embedded channels, called porins, allow the passage of various small water-soluble molecules, such as carbohydrates, amino acids, and metal ions [2,3]. The most abundant and best characterized porin is OmpF.

Enrofloxacin belongs to the class of fluoroquinolones and is a DNA-gyrase inhibitor [4,5,6]. It is active against Gram-negative and Gram-positive bacteria, but it is only used in veterinary medicine [6]. To reach the intracellular target, enrofloxacin must cross the outer membrane first. Evidence for the porin pathway came from the Minimal Inhibitory Concentration (MIC) values of the bacteria that lack the OmpF or OmpC porin [1,4,5,6,7]. 

In previous years, the permeation of various antibiotics across OmpF that has been reconstituted in symmetric planar membranes [7,8,9,10,11,12,13] or reconstituted via the fusion of outer membrane vesicles [13] has been investigated. The application of a transmembrane potential induces an ion current sensitive to modification in the constriction zone. The addition of antibiotics on one or both sides of the chamber will allow them to diffuse to the pore. The permeation of antibiotics into the channel causes an interruption in the ion current. Depending on the affinity for the channel, the residence times range from 100 μs, as in the case of ampicillin, to ms, as in the case of enrofloxacin. In order to distinguish the intrinsic noise from the antibiotic-induced ion current blockage of the channel, we selected enrofloxacin as the model antibiotic, known for its strong interaction with OmpF; In particular, enrofloxacin has an unusually strong affinity to the aspartic acid at position 113 [14]. Yamashita et al. suggested that this position host is also a binding site for divalent ions such as magnesium [14], which could be proved through a comparison of enrofloxacin translocation in magnesium-free buffer and in buffer containing magnesium ions [9,12]. 

In the present study, the focus is on the influence of the different LPS structures on the function of OmpF and the antibiotic translocation. The LPS were isolated from two different deep-rough mutants, *Escherichia coli* strain WBB01 and *Proteus mirabilis* strain R45. Both LPS structures contain the lipid A anchor and a part of the core region [15]. The fatty acid chains are bonded to the diglucoseamine-disaccharide (GlcN). The part of the core region is composed of the two 2-keto-3-desoxyoctonacids (Kdo). The difference between the LPS and the different types of bacteria is the LPS’ additional 4-amino-4-deoxyarabinose (Ara4N). In the case of LPS WBB01, there is no Ara4N, while in case of LPS R45, there is one Ara4N linked to the lipid A and one linked to the first Kdo. Both Ara4N are present with a probability of about 50%, leading to a reduced net charge of −3e_0_. A particular question in this context is the orientation of the channel in the presence of the LPS. Earlier measurements on single channel reconstituted in symmetric membranes revealed an asymmetry in conductance at 0.1 mM KCl and at pH 6 [16]. However, this asymmetry could not be correlated with the physical orientation of the channel. The strong influence of the asymmetric LPS-containing membranes on the intercalation and voltage-dependence of the porin PhoE has been demonstrated in previous research [17].

The major goal of this investigation was to understand the complex three-component system of the asymmetric LPS-containing membranes, the porin OmpF, and the antibiotic, with enrofloxacin as an example. It became clear that the LPS influenced the interaction between the membrane and the OmpF as well as between the membrane and the enrofloxacin. Furthermore, we could demonstrate that the translocation of the enrofloxacin through the membrane was influenced by the number of OmpF in the membrane, and that the deep-rough mutant investigated does not change the constriction of the OmpF.

## 2. Materials and Methods

Chemicals, phospholipids and lipopolysaccharides—In this study, potassium chloride (KCl); magnesium chloride (MgCl_2_); 4-2-hydroxyethyl-1-piperazineethanesulfonic acid (HEPES); potassium hydroxide (KOH) (Sigma Aldrich, Buchs, Switzerland); chloroform and methanol (Merck KGaA, Darmstadt, Germany); phosphatidylethanolamine from *E. coli* (PE); phosphatidylglycerol from *E. coli* (PG); cardiolipin (diphosphatidylglycerol, DPG); and 1,2-diphytanoyl-sn-glycero-3-phosphocholine (DPhPC) (Avanti Polar Lipids, Alabaster, AL, USA) were used. The OmpF was purified by Helge Weingart (Jacobs University, Bremen, Germany) according to a published protocol [10,18]. The lipopolysaccharide was extracted using the phenol/chloroform/petroleum ether method [19], purified, and then lyophilized at the Research Center Borstel. Two types of LPS from deep-rough mutant strains were used, one from the *E. coli* strain WBB01 and one from the *P. mirabilis* strain R45. The enrofloxacin and ampicillin (Fluka Analytical, Steinheim, Germany) were solved in a buffer containing 100 mM KCl, 5 mM MgCl_2_, 5 mM HEPES at pH = 7 to a final concentration of 2 mM (enrofloxacin) and 100 mM (ampicillin). The phospholipid solution (PL) contained a PE:PG:DPG ratio of 81:17:2 [12,20] at a final concentration of 2.5 mg/mL in chloroform. The LPS were solved in chloroform:methanol (9:1), also to a final concentration of 2.5 mg/mL. 1,6-diphenyl-1,3,5-hexatriene (DPH) is a fluorescent dye that was used for the polarisation measurements. It was solved in 96% ethanol to a final concentration of 2 mM and mixed with LPS in a molar ratio of 1:100.

Montal-Mueller membranes—The reconstituted membranes were performed using the Montal-Mueller technique [12,21,22]. Two chambers of a cuvette were separated by a 25 µm thick Teflon foil (Good fellow, Cambridge, UK). The Teflon foil contained a circular aperture with diameters between 60 µm and 120 µm. The Teflon foil was pre-treated with 1% hexadecane in hexane. Once the hexane evaporated, the two chambers were filled with buffer (100 mM KCl, 5 mM MgCl_2_, 5 mM HEPES, pH = 7). The bilayer was formed by adding 10 µL of the LPS to the so-called trans side (connected to the amplifier) and the lipid solutions to the cis (ground electrode) side of the buffer surfaces. Once the organic solvent evaporated, the bilayer could be formed by lowering and raising the buffer levels [20]. The measurements of the single OmpF porin were recorded with an Axopatch 200B amplifier (Axon Instruments, Foster City, CA, USA) connected to Ag/AgCl electrodes (World Precision Instruments, Sarasota, FL, USA). All the measurements were performed at 37 °C. The recorded signal in this particular set-up was filtered using a four-pole low-pass Bessel filter at a frequency of 5 kHz and sampled at 50 kHz using a Digidata 1322A digitizer. The data shown are filtered at 2 kHz and analyzed using the pClamp 10.0 software (Axon Instruments, Foster City, CA, USA). To quantify the permeation of the enrofloxacin, we recorded the ion current fluctuation a few minutes after the addition. To simplify the data analysis, we avoided concentrations above 0.4 mM, which would have caused a double- or multi-channel blockage. For the analysis, we recorded several minutes (>1000 events) and repeated the experiment at least three times.

Polarization measurements—To detect the effect of the enrofloxacin on the phase behavior of LPS we used polarization measurements. DPH as fluorescence probe was inserted into the lipid phase, and the degree of polarization was related to the fluidity of the lipid membrane. The LPS WBB01 vesicles were prepared in 100 mM KCl, 5 mM HEPES, pH = 7. The final concentration of the LPS WBB01 was 0.1 mM and contained 1% 1,6-diphenyl-1,3,5-hexatriene (DPH). The suspension was sonicated for one minute (Branson, Sonifier, Cell disruptor B15) and run through a three-step thermo cycle (30 min at 67 °C and 30 min at 4 °C). Prior to use, we stored the vesicle solution overnight at 4 °C. The enrofloxacin was solved in 0.01% trifluoroacetic acid (TFA) (acidulate with HCl) at a final concentration of 1 M. For the measurements 90 µL of the LPS WBB01, the vesicle solution was mixed with 10 µL of the enrofloxacin solution, and the polarization was measured between 20 °C and 45 °C. All the measurements were performed twice for each preparation, and each sample prepared was measured twice. 

Antibacterial test—The antibacterial activity of the enrofloxacin was tested. Therefore, 96 well plates were used with 90 µL 20 mM HEPES, 150 mM NaCl at pH = 7.4 and 10 µL of bacterial suspension in each well. To determine the minimal inhibitory concentration, the enrofloxacin was solved in 0.01% TFA and added to the bacterial suspension in different concentrations. Controls wells without antibiotics (with bacteria) and wells only filled with buffer were used to obtain the information about full growth and no growth of the bacteria. The prepared plates were closed and incubated overnight at 37 °C. The next day, the extinction of the optical density at 620 nm was measured (Tecan Rainbow, Salzburg, Austria). The concentration of that well, which showed no growth of the bacteria at the lowest antibiotic concentration, was taken as the minimal inhibitory concentration (MIC). For the experiments, two strains of *E. coli* K12 were used: the wildtype strain W3110 and an OmpF deletion mutant W3110ΔompF from Dr. H. Weingart (previously Jacobs University Bremen, now renamed Constructor University) [10,23,24].

## 3. Results

### 3.1. Insertion of OmpF into the Membranes

**Figure 1:**
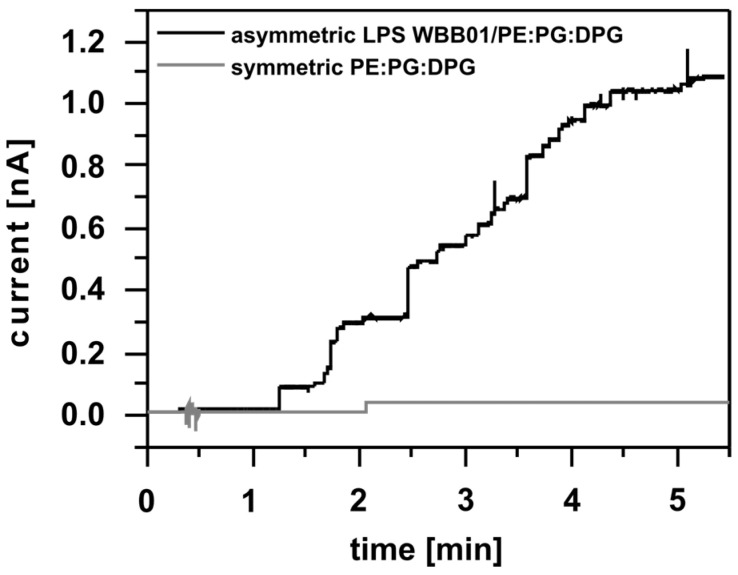
Intercalation of OmpF into symmetric PE:PG:DPG (81:17:2, grey line) and asymmetric LPS WBB01/PE:PG:DPG (81:17:2 [M:M:M], black line) Montal-Mueller membranes. It can be seen that OmpF channels insert faster into asymmetric membranes than symmetric membranes. Buffer: 100 mM KCl, 5 mM MgCl_2_, 5 mM HEPES, pH = 7; T = 37 °C; Clamp voltage: 20 mV; Porin concentration: 8.6 ng/mL.

First, the effect of the LPS WBB01 and LPS R45 on the insertion and function of the OmpF was tested. Asymmetric bilayers were prepared and the OmpF was added to the LPS (amplifier) or phospholipid (ground) side. The results reveal that the porin inserts more rapidly and in larger quantities into the phospholipid side (Figure 1), in contrast to the OmpF, which resulted in almost no insertion into the LPS side during a similar timeframe. This was also observed for PhoE [17] and confirmed the influence of LPS on porin insertion. 

As a control to prove the influence of LPS on porin intercalation, experiments on symmetric phospholipid membranes were performed. OmpF inserted only to a minor extent into symmetric PE:PG:DPG membranes (Figure 1). Note that there was no visible voltage effect on porin insertion.

### 3.2. Oriented Insertion of OmpF into the Membranes

Functional studies often reveal an asymmetry, and thus the orientation of the membrane channel is an important piece of information. A previous study demonstrated the orientation of OmpF in natural *E. coli* outer membranes [25]. A more recent attempt with chemical labelling of a chiral sugar revealed that Maltoporin inserts mainly in symmetric lipid bilayers in an orientation opposite to a natural one [26]. The ion current across OmpF is asymmetric with respect to the applied voltage and thus provides an indication of the degree of orientation of the protein in the membrane [27,28]. A more recent approach introducing OmpF chemical labelling accessible only from one side allowed researchers to conclude the true orientation [29]. For example, if the OmpF is added to the ground side and shows higher ion current at a positive voltage, we follow reference [29] and call this P-OmpF; otherwise, the orientation is called N-OmpF. 

It is observed that the ion current through the cation selective OmpF is slightly lower in PC membranes than in asymmetric LPS WBB01 and LPS R45 membranes. This is likely due to the enhanced concentration of the cationic mobile counter ion cloud around the negatively charged LPS in the asymmetric membrane, which has been nicely demonstrated in pure DPhPC. At low salt concentrations, OmpF in negatively charged DPhPS membranes has a ~15% higher conductance compared with OmpF in neutral DPhPC membranes [28]. Additionally, the conductance was dependent on the pH of the buffer solution as described by Nestorovich et al. [16]. They showed that at low salt concentrations the conductance of OmpF is higher at pH 8 than at pH 3. This also applies to OmpF in LPS-containing membranes. Between LPS WBB01/PE:PG:DPG and LPS R45/PE:PG:DPG, the membranes exhibited no significant differences in the conductance of OmpF.

**Figure 2:**
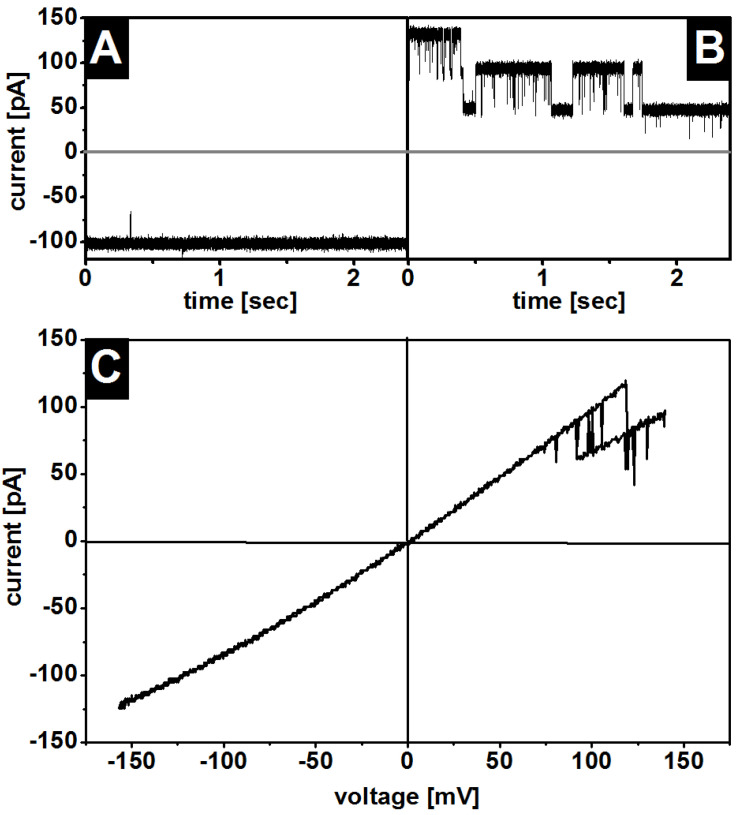
Typical ion current and gating of single OmpF trimer in asymmetric LPS WBB01/PE:PG:DPG membranes. Current trace at a constant clamp voltage (**A**): U = −120 mV; (**B**): U = 120 mV and (**C**) I/U-curve of one OmpF trimer. In this particular orientation (P-OmpF [29]). In this orientation OmpF shows no gating at a negative voltage (**A**): U = −120 mV and (**C**) and gating at a positive voltage (**B**): U = 120 mV and (**C**). Buffer: 100 mM KCl, 5 mM MgCl_2_, 5 mM HEPES, pH = 7; T = 37 °C.

For example, when the OmpF corresponded to the orientation shown in Figure 2A, the application of a negative voltage caused, in all cases, a slightly lower ion current (Figure 2A) than the application of a positive voltage (Figure 2B). When the protein had the opposite orientation (N-OmpF), the results were the other way around. Figure 2C shows the entire I/V asymmetry in the range of −150 mV to 150 mV. 

**Figure 3:**
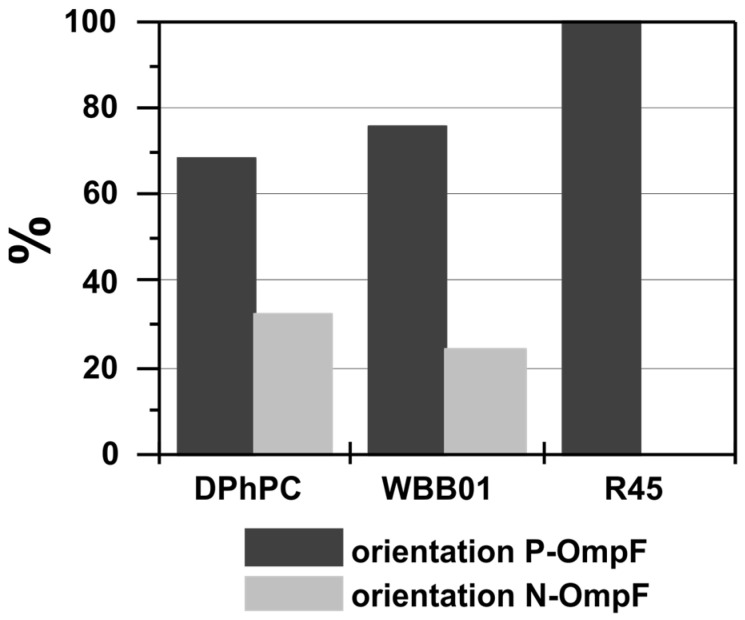
Distribution of the orientation of reconstituted OmpF in various planar membranes. If OmpF is added to the ground side, the P-orientation corresponds to higher positive ion current (black) whereas lower conductance at positive potentials is called N-OmpF (grey) [29]. The first column reports the observation using symmetric DPhPC membranes, the following column the orientation in asymmetric LPS WBB01, and the third LPS R45)/PE:PG:DPG. This was the same for all analyzed channels. In LPS WBB01/PE:PG:DPG and symmetric DPhPC membranes P-OmpF was preferred.

The asymmetry in conductance allows for the determination of the orientation statistics of the OmpF. Around 70% of all the porins in the symmetric DPhPC membranes correspond to the P-OmpF and 30% N-OmpF (Figure 3) in single-molecule recordings. For asymmetric membranes composed of LPS WBB01/PE:PG:DPG with four negative charges and four saccharides on the LPS WBB01, more than 75% of the inserted porins were P-OmpF and less than 25% were N-OmpF (Figure 3). When LPS R45/PE:PG:DPG—which has on average one additional saccharide and an average net charge of −3e0—was used on the LPS R45, the orientation was in all the measurements P-OmpF (Figure 3). Obviously, the LPS structure has an influence or promotes the orientation of the OmpF. The LPS with more sugar groups enhanced the orientation (Figure 3), and the number of sugar groups seems to be more important than the net charge of the respective LPS. However, in bacterial cells, the unfolded OmpF is transported through the inner membrane into the periplasmic space and then into the outer membrane. The secondary and tertiary structure of the pore is developed during the insertion of the protein into the membrane [30]. This brings the long loops to the outside (N-OmpF), whereas a spontaneous insertion favors the opposite orientation (P-OmpF). 

All further results which are shown in the following experiments were generated using the P-OmpF. 

### 3.3. Gating

Closure of OmpF at higher voltage has been observed under various conditions in symmetric membranes. In this study, gating occurred only at positive voltages (Figure 2B), no matter if they were inserted into symmetric or asymmetric membranes. There was almost no gating at negative voltages (P-OmpF) (Figure 2A), and the pores could not be closed at high voltages without breaking the membrane. In contrast, at positive voltages the porins started to close in LPS WBB01/PE:PG:DPG membranes at 106 mV ± 17 mV and in DPhPC membranes at 153 mV ± 17 mV. The differences between the symmetric and LPS-containing membranes under the same conditions suggest that the membrane components have an influence but are not the main cause of the gating. In particular, the transmembrane potential difference within asymmetric membranes might explain the difference [31]. Given our measurements, we may exclude the LPS as the primary cause of the OmpF gating, but the presence of larger LPS molecules in vivo might further reduce the threshold voltage. The biological relevance of the experimentally measured value of −26 mV membrane potential across the bacterial outer membrane remains an open question [32,33]. 

### 3.4. Antibiotic Interaction with Lipid Membranes

**Figure 4:**
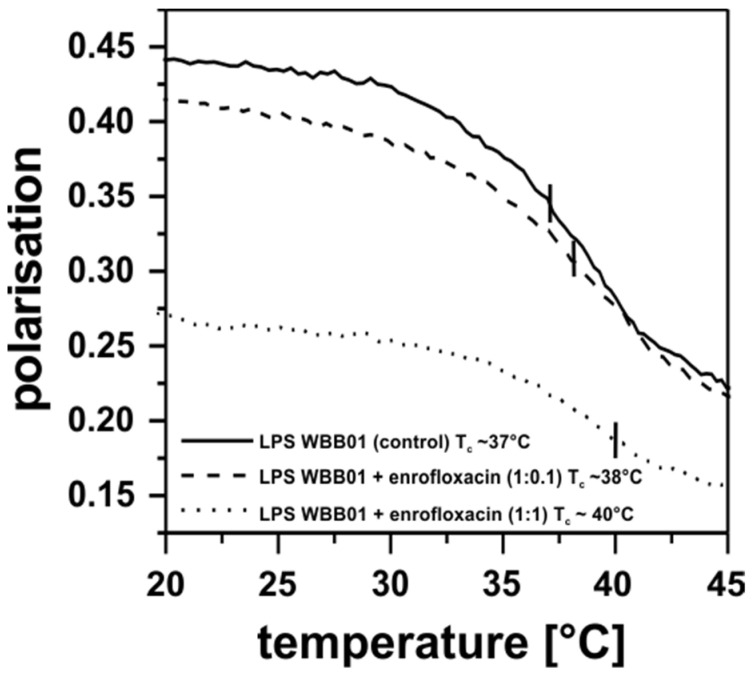
Influence of different enrofloxacin concentrations on the phase transition temperature of LPS WBB01 analyzed using polarisation measurements. Both concentrations induced a fluidization of LPS WBB01 at low temperatures. LPS WBB01 concentration: 0.01 mM; DPH: 1%; concentration of enrofloxacin stock solution: 1 mM; buffer: 100 mM KCl, 5 mM HEPES, pH = 7.

The polarisation measurements of the LPS WBB01 in the presence of the enrofloxacin indicated a small effect on the phase transition temperature of the fatty acid chains. The LPS WBB01 membrane was more fluid in presence of the enrofloxacin at a low temperature and at a molar ratio of 1:0.1 (LPS WBB01:enrofloxacin). At higher concentrations (1:1), the effect was strong over the entire temperature range (Figure 4). Thus, enrofloxacin intercalates into the hydrophobic area of the membrane and can strongly influence the electrical and mechanical properties of the membrane. It could be suggested that the uptake in antibiotics by the bacterial membrane also play a role in bacteria killing; however, it could be shown that bacteria that lack the outer membrane porins are more resistant to antibiotics. Although the enrofloxacin can interact with the membrane and change its fluidity, it is unlikely for it to pass through the membrane in large amounts. It is interesting to note that this is in agreement with an earlier observation that suggested a higher permeability for LPS from deep-rough mutant bacteria compared with the fully extended LPS [33]. 

### 3.5. Antibiotic Interaction with OmpF

**Figure 5:**
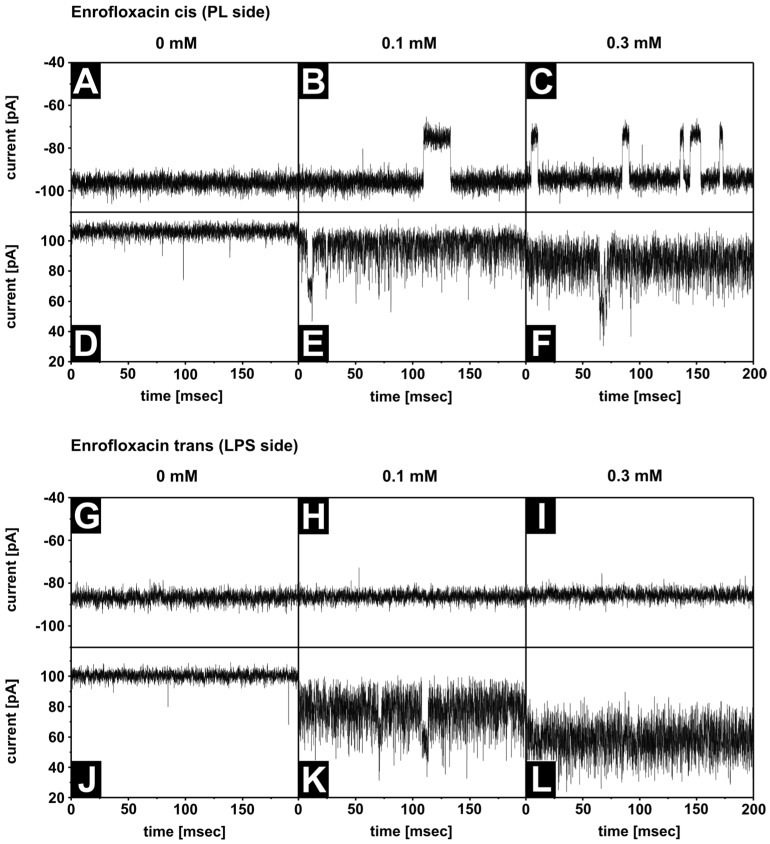
Current traces of one OmpF trimer in asymmetric LPS WBB01/PE:PG:DPG membranes with and without different enrofloxacin concentrations. As controls, the OmpF current traces were measured without enrofloxacin at U = −100 mV (**A**–**C**,**G**–**I**) and U = +100 mV (**D**–**F**,**J**–**L**). Enrofloxacin blocked the pore concentration dependent (0.1 mM and 0.3 mM) if it was added to the cis side at U = −100 mV (**B**,**C**) and show an increased noise and a lower current level than at U = 100 mV (**E**,**F**). If enrofloxacin was added to the trans side, no concentration dependent blockage was seen (**H**,**I**,**K**,**L**), but the noise increased and the current decreased at U = 100 mV (**K**,**L**). Buffer: 100 mM KCl, 5 mM MgCl_2_, 5 mM HEPES, pH = 7; T = 37 °C.

Previous measurements of OmpF reconstituted in symmetric DPhPC membranes revealed ion current blockages in the presence of antibiotics [12,34,35]. The residence time decreases with increasing temperature. In order to resolve the blockage at 37 °C, we selected the enrofloxacin with residence times at room temperature, around ms. It should be noted that the stability of an asymmetric LPS/PL membrane requires the presence of Mg^2+^ ions in the buffer. Previous measurements have been performed in Mg^2+^ free solutions. To analyze the influence of lipopolysaccharides on the antibiotic interaction with the OmpF, we reconstituted single OmpF channels in asymmetric bilayers, which requires Mg^2+^. Figure 5 summarizes the current traces of a single OmpF trimer in an asymmetric LPS WBB01/PE:PG:DPG membrane at +100 mV/−100 mV under various conditions. Under all the conditions, the number of blockages of the ion current through the OmpF increases with an increasing enrofloxacin concentration (Figure 5A–C), if added to the cis side at a negative voltage. 

**Figure 6:**
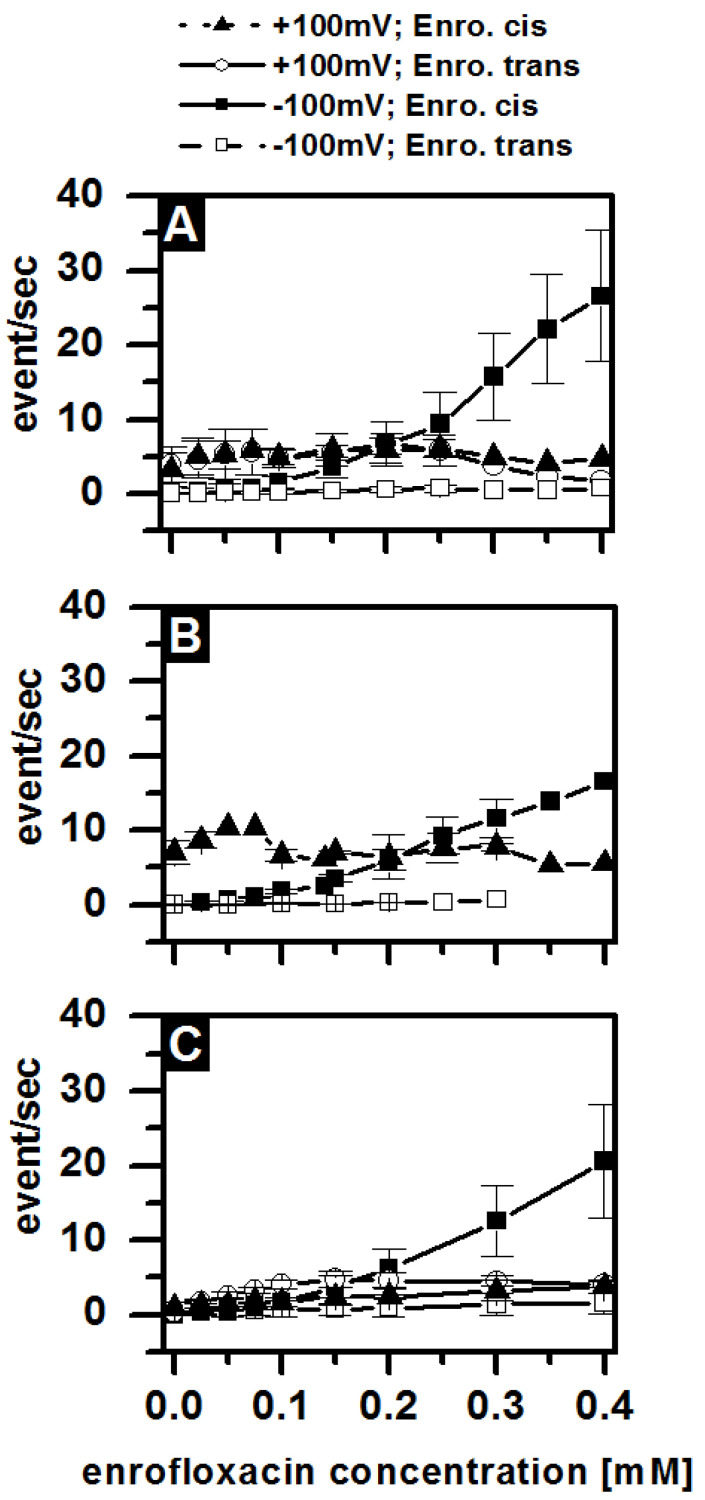
Enrofloxacin blockages per second in dependence of the enrofloxacin concentration. The composition of the asymmetric LPS (LPS WBB01 and LPS R45)/PE:PG:DPG) membranes composed either of (**A**) LPS R45 or (**B**) of LPS WBB01 had no effect on the enrofloxacin blockage compared to (**C**) the symmetric DPhPC membrane. Buffer: 100 mM KCl, 5 mM MgCl_2_, 5 mM HEPES, pH = 7; T = 37 °C; Clamp voltage: 100 mV. The data is based on >three independent recordings of several minutes.

These blockages indicate the interaction of the enrofloxacin with the OmpF, and they show that one or more pores of one OmpF trimer are blocked and a reduced current or no current is measurable. The blockage could correspond to a complete translocation of an enrofloxacin molecule through the pore or a temporary blockage without translocation. The residence time of the antibiotic in the OmpF channel [9,12,13] was analyzed in the different membranes and was about 8 ms and independent of the membrane composition (Table 1). The number of blockages per second was also almost the same in the LPS- and DPhPC-containing membranes (Figure 6). 

In Figure 6, the results of the analysis of the long blockages are shown. In contrast, no blockages were detectable at a negative voltage when the enrofloxacin was added to the trans side (Figure 5G–I). At a positive voltage, the noise level increased with the enrofloxacin concentration dramatically and independent of the side of the addition (Figure 5D–F,J–L). The current decreased with increasing enrofloxacin concentrations. Similar effects were also detectable in other LPS-containing membranes and in symmetric DPhPC membranes. The reduced ion current and increased noise level result from blockages [12], which were not resolvable within the time resolution of the electrical setup. Our data support the existence of two binding sides, or two different translocation pathways, of the enrofloxacin in the OmpF molecules. Both depend on the side of the enrofloxacin addition, the polarity of the applied external voltage, and the presence of Mg^2+^ ions.

**Figure 7:**
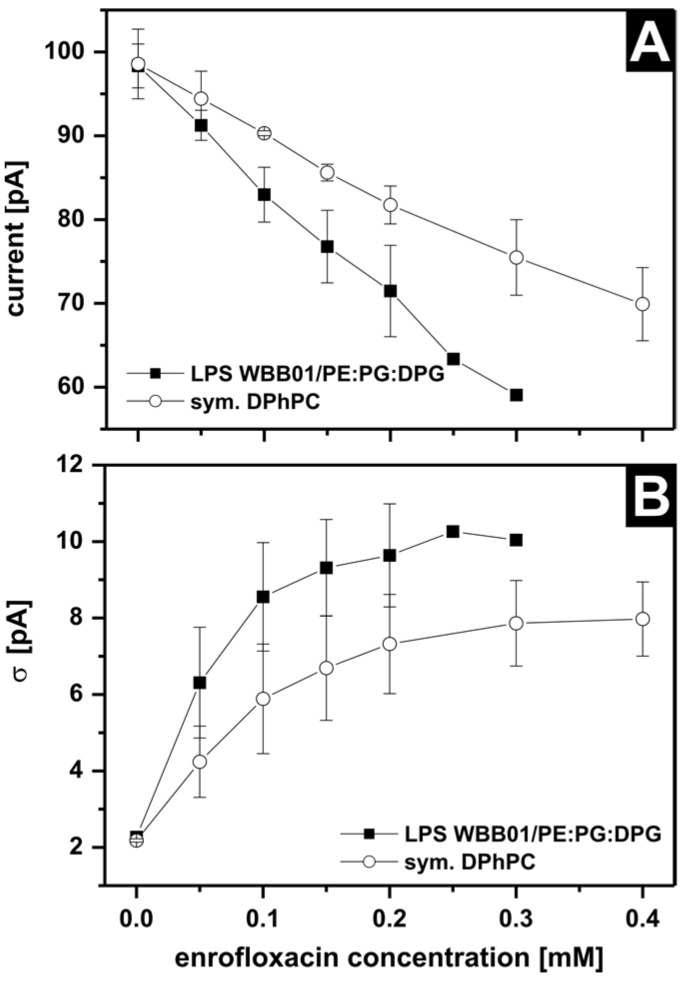
Changes in (**A**) the current of one OmpF trimer by different enrofloxacin concentrations on the trans side at U = 100 mV in different membranes (WBB01/PE:PG:DPG; symmetric DPhPC) and (**B**) changes of the noise indicated by the half width of the Gaussian distribution σ. Buffer: 100 mM KCl, 5 mM MgCl_2_, 5 mM HEPES, pH = 7; T = 37 °C; Clamp voltage: 100 mV; Enrofloxacin on the trans side.

To analyze the reduction in current and the increase in noise, histograms of the current traces were performed and fitted by Gaussian distribution. Figure 7A shows the almost linear decrease in the ion current of the asymmetric LPS WBB01/PE:PG:DPG and the symmetric DPhPC membranes by increasing the enrofloxacin concentrations. Figure 7B shows the half width (σ) of the Gaussian distribution, which reflects the increased noise. The noise increased with increasing enrofloxacin concentrations up to a saturation concentration of about 0.2 mM. For the analysis, all single data points were normalized to a concentration of 1 mM and were analyzed by a *t*-test to determine the significance. The significance for the two curves (Figure 7A) was *p* = 0.001. Although the noise was increased, some long blockages (closure of the pore) were detectable. However, these blockages were enrofloxacin concentration independent. 

In contrast, in the absence of Mg^2+^, Mahendran et al. [10] (2010) showed that there were no differences in blocking the pore through enrofloxacin, independent of whether the antibiotic was added to the cis or trans side. Molecular modelling revealed that Mg^2+^ has the same binding side [27] on the amino acid residue D113 in the constriction zone of the pore as enrofloxacin. If the D113 was replaced by an asparagine, the interaction of the enrofloxacin with the pore was changed [9]. Control experiments using symmetric DPhPC membranes revealed that the presence of magnesium reduced the number of blackages by 50%. The reduced current by the increased enrofloxacin concentration and the increased noise level was caused by the magnesium ions [12].

### 3.6. Antimicrobial Test

Microbiological tests were performed to confirm the influence of the OmpF on the minimal inhibition concentration (MIC) of the enrofloxacin. The results showed that the MIC values of enrofloxacin in the OmpF deletion mutants *E. coli* strain W3110 ΔompF was about 50 ng/mL, and in the wild type strain W3110 30 it was ng/mL. These results show that OmpF is important in the uptake of enrofloxacin. Cohan et al. described OmpF deletion mutants as having MIC values four to eight times higher than those of wild type bacteria for other fluoroquinolones [36]. The experiments with an OmpF deletion mutant showed that about 60% more enrofloxacin was needed to kill the mutant than the wild type strain. The bacteria may compensate for the deleted porins, and the enrofloxacin may permeate through other channels. 

## 4. Discussion

**Figure 8:**
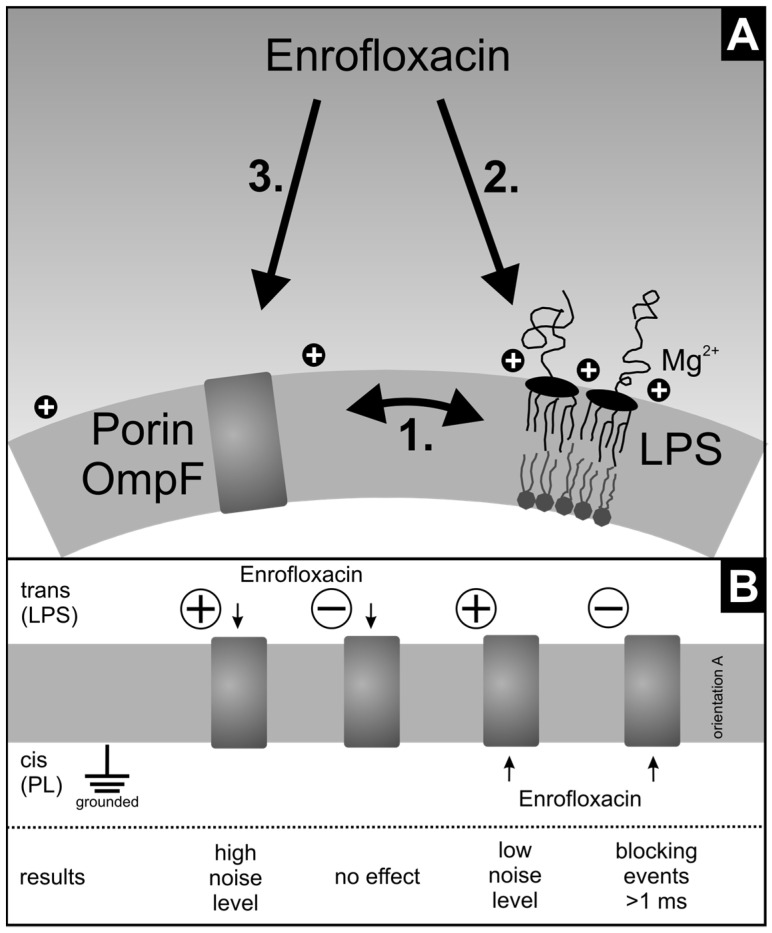
(**A**) Schematic diagram of the different components involved in the uptake of the antibiotic enrofloxacin through the porin OmpF embedded in the outer membrane of Gram-negative bacteria. (1.) LPS and OmpF: intercalation, orientation, and gating of OmpF are influenced by LPS; (2.) enrofloxacin and LPS: enrofloxacin influenced the fluidity of LPS membranes; (3.) enrofloxacin and OmpF: blockage of OmpF depends on lipid composition, clamp voltage, side of addition, and presence of magnesium. Note: In bacteria porin, insertion is promoted by the BAM complex. In contrast to spontaneous insertion, the chaperon insertion results in a long-loop outside [29]. In addition, the inside has a negative Donnan potential [32]. (**B**) Schematic diagram of the enrofloxacin interaction with OmpF at different clamp voltages, as indicated by (−) and (+), and the different sides of enrofloxacin addition.

A particular step in the action of antibiotics is their translocation across the outer cell wall to reach the target. In order to evaluate possible rate limiting steps we characterize the translocation through OmpF in the presence of an asymmetric LPS-phospholipid membrane. Figure 8 gives an overview of the possible models used in this work.

With the appearance of new antibiotics, new resistant bacteria strains also appear. Because of their short life cycle, bacteria with mutations that make them resistant to new antibiotics spread quite fast. To develop new antibiotics, it is necessary to understand the uptake of antibiotics by the bacterial cell. It is known that porins in the outer bacterial membrane are involved in the uptake of antibiotics. The results show that the LPS has a strong influence on the insertion, orientation, and gating of the OmpF and likely also on the other porins. Compared to the phospholipids, the LPS has a larger polar head group, which implies a highly negative change density and a steric repulsion. Also, the stabilization effect of the Mg^2+^ ions might provide a dense layer that is difficult for the porins to penetrate and thus reach the hydrophobic membrane. Another aspect could be the asymmetry of the membrane itself. The asymmetry might provide a favorite curvature promoting the insertion from the opposite side. 

The orientation of the OmpF in bacterial cell membranes was opposite to the favorite orientation found in the spontaneous insertion in artificial membranes. All the analyzed single channel measurements showed a dominant orientation in the LPS R45/PE:PG:DPG membranes. The OmpF inserts only into asymmetric membranes when it was added to the phospholipid side and not when it was added to the LPS side. This was also shown for PhoE [17], and confirms the strong influence of LPS on porin insertion. Aside from LPS, it has been demonstrated that different types of phospholipids have a stronger or weaker binding affinity to OmpF [37]. The negatively charged phosphatidylglycerol, for example, binds less strongly than phosphatidylcholine or phosphatidylethanolamine. 

One point which is, to date, unclear is the molecular and physical origin of OmpF gating. It is known that gating is dependent on the experimental conditions and the purification of the protein. The gating property is very reproducible for individual porins and shows only a small scattering within a batch. However, samples from different batches may exhibit substantially different behaviors. Different research groups described a wide range of threshold voltages (Vc) for OmpF, such as from 90 mV [38] up to between 150 and 170 mV [2,39,40]. Robertson and Tieleman summarized the different hypotheses which can lead to the porin gating [41]. Experimental data show that the L3 loop which constricts the size of the pore is not the main factor in porin gating. There are also other factors, such as specific residues in the pore, the other loops on the periplasmic side of the channel, and the beta-barrel itself. 

Independent of the voltage gating, we could show that the enrofloxacin blocked the channel at negative and positive voltages. We found no significant differences in the enrofloxacin blockage of OmpF in different membranes. But the interactions at positive voltages are much faster [12] than the interactions at negative voltages. 

Even if the results also show an influence of enrofloxacin on LPS membrane fluidity, the assumption is that it is not the main mechanism of antibiotic uptake. This is in agreement with the results published by Mestres et al., 1994 [42], who demonstrated that enrofloxacin has no strong interactions with phospholipids when incubated with liposomes and that it changes the fluidity of the polar head group. This weak interaction on the membrane surface indicates that the enrofloxacin uses the porin to enter the bacteria and not the hydrophobic part of the membrane. It is possible that the change in membrane fluidity also changes the properties of the OmpF and therefore the uptake of the enrofloxacin. 

## 5. Conclusions

In summary, it has been shown that the LPS of deep-rough mutants have an influence on the insertion of the OmpF porin. Furthermore, the antibiotic enrofloxacin has an influence on the LPS/PL membrane properties. However, the LPS layer has only a small effect on the antibiotic translocation through the OmpF. For the latter, the rate-limiting step is the interaction inside the channel.

## Figures and Tables

**Table 1:** Residence time (τ) of enrofloxacin in OmpF incorporated into different LPS-containing membranes and DPhPC membranes.

		τ (−100 mV)	τ (+100 mV) (Only the Long Blockages)
LPS R45/PL	enrofloxacin cis	8.2 ms ± 5.3 ms	1.1 ms ± 0.4 ms
	enrofloxacin trans	no events	1.6 ms ± 0.5 ms
LPS WBB01/PL	enrofloxacin cis	5.3 ms ± 2.4 ms	0.9 ms ± 0.3 ms
	enrofloxacin trans	no events	not resolvable
DPhPC/DPhPC	enrofloxacin cis	10.5 ms ± 3 ms	1.1 ms ± 0.1 ms
	enrofloxacin trans	no events	0.8 ms ± 0.2 ms

## Data Availability

Not applicable.
